# Creation of a National Emergency Medicine Medical Education Journal Club

**DOI:** 10.7759/cureus.54092

**Published:** 2024-02-12

**Authors:** Jessica Pelletier, James Ahn, Andrew Golden, Caroline Astemborski, Michelle D Lall, Albert Kim, Sara Dimeo

**Affiliations:** 1 Emergency Medicine, Washington University School of Medicine, St. Louis, USA; 2 Emergency Medicine, University of Chicago, Chicago, USA; 3 Emergency Medicine, University Hospitals Cleveland Medical Center, Cleveland, USA; 4 Emergency Medicine, Prisma Health, Greenville, USA; 5 Emergency Medicine, Emory University School of Medicine, Atlanta, USA; 6 Emergency Medicine, Dignity Health, Chandler, USA

**Keywords:** community of practice, journal club, medical education fellowship, medical education, emergency medicine

## Abstract

Background

There are a relatively limited number of emergency medicine (EM) medical education (MedEd) fellowships with few trainees at each program, creating barriers to local collaboration and networking. While best practices for developing MedEd journal clubs exist, there has not been an established national EM MedEd journal club. To address this need, we created a national journal club, the Council of Residency Directors (CORD) MedEd Journal Club (MEJC), to facilitate collaboration and networking opportunities by providing a synchronous online journal club.

Objectives

Our primary objective was to create a network for collaboration across geographical barriers to form a virtual community of practice (CoP) around the shared domain of evidence-based MedEd. Our secondary objective was to improve MedEd fellows’ knowledge, skills, and attitudes surrounding MedEd research. Tertiary objectives included (1) broadening fellow exposure to key topics within MedEd, (2) describing how to develop scholarly work within MedEd, and (3) filling a perceived need for building a national MedEd virtual CoP.

Curricular design

The concept and objectives of the CORD MEJC were introduced to fellows and fellowship directors through a national listserv in March of 2022. Fellows volunteered to lead virtual sessions via Zoom on a monthly basis. Session fellow leaders independently chose the topics and were asked to submit two to three journal club articles discussing the topic at least two weeks in advance of each session. No topics were repeated throughout the academic year.

Impact/effectiveness

Our quality improvement survey results indicated that the CORD MEJC is meeting its primary and secondary objectives. Survey results will be utilized as part of a continuous quality improvement initiative to enhance our program structure and curricula for the 2023-2024 academic year.

## Introduction

The practice of journal clubs at academic medical centers is well established, beginning almost 150 years ago with the introduction by Sir William Osler at McGill University [1]. Journal clubs have grown in popularity across all specialties and serve as forums for ongoing medical education (MedEd) and knowledge-sharing. Journal clubs have also been shown to allow for critical evaluation of the scientific literature as well as keeping up to date on current research [1,2].

MedEd fellowships allow for subspecialization in education theory, andragogy, and MedEd research. As of 2020, there were 45 emergency medicine (EM) MedEd fellowships in the United States, with a total of 91 fellows [3]. Given that there are relatively few MedEd fellowships, networking with peers and like-minded experts is important for continued collaboration and the development of communities of practice (CoP). The CoP learning theory posits that groups of individuals with shared interests gather together in response to a perceived need. A CoP requires a domain (shared interest), a community (which grows out of the domain), and a practice (formation of outputs that benefit the group). Novice members participate in the CoP via a process referred to as "legitimate peripheral participation," in which they contribute to the community and practice in small ways, growing via mentorship into full members of the CoP [4].

There are relatively few MedEd fellowships, and each program houses few trainees, potentially serving as a barrier to local collaboration and networking opportunities. Despite the incorporation of journal clubs in all specialties and academic centers, there has been limited scholarship exploring MedEd journal clubs. While a prior publication described the best practices for starting MedEd journal clubs [5], to our knowledge, a national EM MedEd journal club has not been established, and no other work has studied outcomes for EM journal clubs in general. Other areas in EM, such as simulation, have previously developed similar national journal clubs [6].

Our national journal club seeks to provide collaboration and networking opportunities across geographical barriers by providing a synchronous online journal club not otherwise feasible. The goals of the journal club include the development of a working knowledge of MedEd, applying critical appraisal of the literature to scholarly work, and creating a network for collaboration across geographical barriers in order to form a virtual CoP around the shared domain of evidence-based MedEd.

Study aim/objectives

The primary objective of this study is to assess the impact of the Council of Residency Directors (CORD) MedEd Journal Club (MEJC) on MedEd fellows' self-reported ability to share ideas and network outside of their institution. A secondary objective is to improve fellows' self-reported knowledge related to MedEd research methods. We also conducted a programmatic assessment to evaluate if the CORD MEJC is an effective tool for, (1) broadening fellow exposure to key topics within MedEd, (2) describing how to develop scholarly work within MedEd, and (3) filling a perceived need for building a national MedEd virtual CoP.

## Materials and methods

Theoretical framework

Our curriculum design is based on the theoretical framework of the System of Profound Knowledge, which theorizes that comparing observations with predictions makes quality improvement (QI) systematic [7]. The System of Profound Knowledge uses a cycle referred to as “Plan-Do-Study-Act” (PDSA) to conduct continuous QI [6]. These stages of the cycle include (1) plan, in which a problem is identified and a potential solution is devised to address it; (2) do, in which a solution is implemented to address the problem; (3) study, wherein a systematic analysis is conducted to assess whether the solution appropriately addressed the problem; and (4) act, in which necessary changes that were identified during the study stage are used to enter the next PDSA cycle to address the problem [7]. Our assessment of our journal club intervention using the PDSA cycle of QI allows us to review our program structure during the next academic year. 

Curricular design

The concept and objectives of the CORD MEJC were introduced to fellows and fellowship directors through a national listserv in March of 2022. To receive emails through this listserv, one must (1) be a CORD member and (2) apply to join the CORD MedEd Fellowship CoP (which only admits emergency MedEd fellows and faculty). A sign-up using Google Sheets® was created and disseminated via the listserv that included the dates of each session and allowed each fellow to input their name, program, and contact information. A suggested list of MedEd topics was provided at the bottom of the spreadsheet. This list was created by the CORD MEJC coordinator, who is an attending physician in academic EM who completed a fellowship in MedEd. Volunteers interested in presenting selected one of the suggested topics or emailed the CORD MEJC coordinator for approval of another topic of their choosing.

For ease of coordination, the sessions were held on the first Thursday of every month with flexibility on exact timing. Session fellow leaders independently chose the topics and were asked to submit two to three journal club articles discussing the topic at least two weeks in advance of each session. No topics were repeated throughout the academic year. Topics discussed during these sessions are listed in Table 1. The session coordinator sent out Zoom links and articles through the CORD MedEd listserv one week before the sessions. An attending physician from the presenting fellow’s institution, typically the MedEd fellowship program director, was present to help supervise the presentation by the fellow during each session. The CORD MEJC coordinator was also present during each session to help moderate the discussion.​​​​​​ ​​​​​​ ​​​​​​

**Table 1:** The 2022-2023 CORD MEJC topics CORD, Council of Residency Directors; GME, graduate medical education; MedEd, medical education; MEJC, Medical Education Journal Club

Topic
Introduction to CORD MEJC, How to Get Your MedEd Research Project From "Pen" to "Paper"
The National Clinical Assessment Tool (NCAT) for Medical Students in Emergency Medicine
Deconstructing Racism...Guiding Principles on Inclusive Curriculum Design
Learning Analytics
Rural Rotations in Emergency Medicine
Bedside Teaching
Remediation in GME
Non-cognitive Skill Assessment
High Versus Low Fidelity Simulation

Research design

After every CORD MEJC session, participants were asked to provide formative feedback to their fellow session leaders on their presentation of the article. Participation in these surveys was voluntary and without compensation. The results of these post-session surveys informed the questions used for the end-of-the-year program evaluation survey, the results of which are presented in this article.

During the CORD 2023 conference, the study authors met and developed a list of survey questions designed to evaluate the study objectives (which had been previously defined but were formally agreed upon by consensus among this group). Survey questions were developed through rounds of repeated editing, and several academic EM physicians from each authoring institution reviewed the survey questions for clarity. At the end of the academic year, the survey to assess the curriculum was sent to the CORD MEJC participants to determine the overall effectiveness in regard to the journal articles/research topics reviewed and the networking opportunities provided. The Institutional Review Board (IRB) at Washington University in St. Louis waived the need for IRB approval, given that this was a quality improvement initiative.

The survey was created with attention to Messick's framework for providing evidence for validity [8]. An iterative process of review by a group of expert medical educators was used to create the survey, using guidelines outlined in Academic and Fowler's Study Methods [9,10]. After initial development, the author group performed a read-aloud followed by a pilot of the survey. Any items that were determined to be irrelevant or confusing were deleted and/or had wording changed for clarity purposes.

Faculty and fellow surveys (Table 2) were divided into three sections. Section 1 included information about the fellowship program setting and inherent educational opportunities available within the fellowship, as well as the engagement of the participant in our MEJC during the 2022-2023 academic year. Section 2 included Likert scale ratings (scale of 1-5) regarding the usefulness of our MEJC for meeting its intended program objectives (please see the “Study aim/objectives” section). Lastly, Section 3 involved free response questions asking for suggestions to improve the MEJC over the 2023-2024 academic year.

**Table 2:** CORD MEJC Fellow and Faculty Survey Questions, 2022-2023 CORD, Council of Residency Directors; GME, graduate medical education; MedEd, medical education; MEJC, Medical Education Journal Club

Respondent Group	Section	Survey Questions
Fellow	1 – Information about fellowship program setting and inherent educational opportunities available within the fellowship; CORD MEJC engagement	What is your geographic region? Answer choices: Northeast, South, Midwest, West
		What is the name of your affiliated institution? Free response
		What type of institution are you affiliated with? Answer choices: Academic, Community, County
		What year in your fellowship are you? Answer choices: First year of a one-year fellowship, First year of a two-year fellowship, Second year of a two-year fellowship
		Does your fellowship offer a degree? Answer choices: Yes, No
		If you selected that your fellowship offers a degree, please list which one. Free response
		Does your fellowship have a particular education “niche”? (for example, simulation, research, education technology) Answer choices: Yes, No, Unsure
		If your fellowship has a particular education “niche," please describe it here. Free response
		In the past year, how often did you independently read MedEd journals? Answer choices: Never, Rarely, Quarterly, Monthly, 1-2 Times Monthly, Weekly
		If you read MedEd journals, which journals do you read? (select all that apply) Answer choices: Academic Emergency Medicine Education & Training, Academic Medicine, Graduate Medical Education, Journal of Education & Teaching - Emergency Medicine (JETem), Medical Education, Medical Teacher, Teaching and Learning in Medicine, Western Journal of Emergency Medicine (West JEM), Other
		If you selected "Other" journals, please list here. Free response
		In the past year with your fellow(s), what medical education topics did you cover outside of the CORD MEJC? (select all that apply) Answer choices: Assessment and feedback; Competency-based medical education; Curriculum design; Diversity/inclusion; Ed tech; E-learning, informatics, multimedia; Education research/best practices; Ethics and professionalism; Faculty development; Resident as teacher; Simulation; Social media; Teaching and learning strategies; Trainee recruitment and selection; Other
		If you selected "Other" topics outside of the CORD MEJC, please list here. Free response
		Which CORD MEJC did you attend this year? Answer choices: Introduction to CORD MEJC, How to Get your MedEd Research Project from "Pen" to "Paper;” The National Clinical Assessment Tool (NCAT) for Medical Students in Emergency Medicine; Deconstructing Racism...Guiding Principles on Inclusive Curricular Design; Learning Analytics; Rural Rotations in Emergency Medicine; Bedside Teaching; Remediation in GME; Non-cognitive Skill Assessment; I don’t remember; None
		What challenges served as barriers to you attending CORD MEJC sessions this year? Free response
		How many medical education research abstracts would you estimate you have published in the past year? Answer choices: 0, 1-3, 4-6, 7-9, 10 or more
		How many medical education research articles have you published in the past year? Answer choices: 0, 1-3, 4-6, 7-9, 10 or more
	2 – Likert scale ratings. Please rate the below questions to the best of your ability, where 1 is strongly disagree and 5 is strongly agree.	Please rate your level of agreement with the following statement: "I feel that I am ready/able to perform medical education research”
		The CORD MEJC sessions have directly improved my knowledge of research methods in medical education
		The CORD MEJC sessions have directly improved my ability to perform medical education research
		The CORD MEJC sessions have directly improved my desire to perform medical education research
		The CORD MEJC sessions have led to networking opportunities otherwise unavailable to me in my fellowship
	3 – Free response questions	Describe ways in which you utilized information from the journal club to improve your scholarly work in medical education research
		Please include any other comments you have about the journal club, suggestions for improvement, or future ideas
Faculty	1 – Information about fellowship program setting and inherent educational opportunities available within the fellowship; CORD MEJC engagement	What is your geographic region? Answer choices: Northeast, South, Midwest, West
		What is the name of your affiliated institution? Free response
		What type of institution are you affiliated with? Answer choices: Academic, Community, County
		What is/are your current role(s) in your medical school or department? (select all that apply) Answer choices: Dean, Associate Dean, Vice Chair of Education, Program Director, Assistant or Associate Program Director, Fellowship Director or Assistant Fellowship Director, Clerkship Director or Assistant Clerkship Director, Clinical Faculty, None of the above/not applicable, Other
		If you selected "Other" role(s), please list here. Free response
		Do you currently have (a) medical education fellow(s) within your department? Answer choices: Yes, No
		Does your fellowship have a particular education “niche"? (for example, simulation, research, education technology) Answer choices: Yes, No, Unsure
		If your fellowship has a particular education “niche," please describe it here. Free response
		Do you currently have an MEJC within your department/hospital/medical school? (separate from a traditional clinically focused journal club) Answer choices: Yes, No
		How often did you review MedEd journal articles with your fellow(s) the previous academic year? Answer choices: We did not review MedEd journal articles, As topics arise, Quarterly, Monthly, Bimonthly, Weekly
		If you read MedEd journals, which journals do you read? (select all that apply) Answer choices: Academic Emergency Medicine Education & Training, Academic Medicine, Graduate Medical Education, Journal of Education & Teaching - Emergency Medicine (JETem), Medical Education, Medical Teacher, Teaching and Learning in Medicine, Western Journal of Emergency Medicine (West JEM), Other
		If you selected "Other" journals, please list here. Free response
		In the past year with your fellow(s), what medical education topics did you cover outside of the CORD MEJC? (select all that apply) Answer choices: Assessment and feedback; Competency-based medical education; Curriculum design; Diversity/inclusion; Ed tech; E-learning, informatics, multimedia; Education research/best practices; Ethics and professionalism; Faculty development; Resident as teacher; Simulation; Social media; Teaching and learning strategies; Trainee recruitment and selection; Other
		If you selected "Other" topics outside of the CORD MEJC, please list here. Free response
		Which CORD MEJC did you attend this year? Answer choices: Introduction to CORD MEJC, How to Get your MedEd Research Project from "Pen" to "Paper;” The National Clinical Assessment Tool (NCAT) for Medical Students in Emergency Medicine; Deconstructing Racism...Guiding Principles on Inclusive Curricular Design; Learning Analytics; Rural Rotations in Emergency Medicine; Bedside Teaching; Remediation in GME; Non-cognitive Skill Assessment; I don’t remember; None
		What challenges served as barriers to you attending CORD MEJC sessions this year? Free response
	2 – Likert scale ratings. Please rate the below questions to the best of your ability, where 1 is "strongly disagree" and 5 is "strongly agree".	The CORD MEJC content was a valuable supplement to the scholarly component of my fellowship program
		The CORD MEJC content was a valuable way to build a virtual community of practice to supplement my fellowship program
		The CORD MEJC sessions have led to networking opportunities otherwise unavailable for my fellows
	3 – Free response question	Please include any other comments you have about the journal club, suggestions for improvement, or future ideas

The survey was created in Google Forms and disseminated via the CORD MedEd Fellowship CoP listserv, with reminders sent biweekly via WhatsApp to the CORD MEJC group chat. Survey results were collected from April 27, 2023, to June 1, 2023. The names and email addresses of the survey participants were not collected. Demographic information (institution and region) was collected for the purpose of identifying the national impact of the CORD MEJC. The "Summary" feature in Google Forms was used to assess the overall response data for the fellow cohort and the faculty cohort of respondents.

We included all surveys completed by respondents in the final data analysis, including those with incomplete data. Google Forms provided immediate aggregation of survey responses from respondents by question number. We report descriptive statistics for items with discrete answer options. Likert scales were translated to ordinal numbers for analysis. We report the proportions of respondents as percentages, taking into account missing data points.

 

 

Research Desig

## Results

A total of 65 individuals were members of the CORD MedEd listserv during the 2022-2023 academic year; data are not available as to how many of these individuals were fellows versus faculty. Eight MedEd fellowship faculty and seven MedEd fellows participated in the survey, yielding a 23% response rate. Faculty respondents were located across all four geographic regions (Northeast, South, Midwest, and West) at eight institutions, while fellow respondents were located in three geographic regions (Northeast, South, and Midwest) at six institutions. One fellow did not name their institution; however, respondents included fellows from six academic programs, one community program, and one county program, totaling 10 unique institutions represented between the faculty and fellow respondents.

The majority of fellows (71.4%; 5 out of 7) indicated that the CORD MEJC was helpful for networking outside of their fellowship, meeting the primary objective. The majority of fellows (85.7%; 6 out of 7) reported that their MedEd fellowship offered a degree (83.4% Master of Health Professions Education (MHPE) or Master of Education in the Health Professions (MEHP); 16.7% allowed the choice of MHPE or Master of Science in Education (MsED)). Less than half of the fellows indicated that the CORD MEJC directly improved their knowledge of research methods (42.9%; 3 out of 7) and desire to perform MedEd research (42.9%; 3 out of 7). More than half reported that the CORD MEJC improved their ability to perform MedEd research (57.1%; 4 out of 7) (secondary objective and tertiary objective 2, Table 3). However, 28.6% of respondents indicated that they found the CORD MEJC unhelpful in improving their MedEd research skills (tertiary objective 2, Table 3).

**Table 3:** Fellow perceptions of the CORD MEJC Likert scale (1-5): 1 = strongly disagree, 2= disagree, 3 = neither agree nor disagree, 4 = agree, and 5 = strongly agree. Each box indicates the number of respondents out of the total number of respondents who selected that Likert scale score. The fraction is also shown as a percentage. CORD, Council of Residency Directors; MEJC, Medical Education Journal Club; N/A, not applicable.

Fellow Survey Question	Likert Scale Score				
	1	2	3	4	5
The CORD MEJC sessions have directly improved my knowledge of research methods in MedEd (i.e., knowledge evaluation)	N/A	N/A	4/7 (57.1%)	3/7 (42.9%)	N/A
Please rate your level of agreement with the following statement: “I feel that I am ready/able to perform MedEd research” (i.e., skills evaluation)	N/A	2/7 (28.6%)	1/7 (14.3%)	4/7 (57.1%)	N/A
The CORD MEJC sessions have directly improved my desire to perform MedEd research (i.e., attitudes evaluation)	N/A	1/7 (14.3%)	3/7 (42.9%)	3/7 (42.9%)	N/A
The CORD MEJC sessions have led to networking opportunities otherwise unavailable to me in my fellowship	N/A	N/A	2/7 (28.6%)	4/7 (57.1%)	1/7 (14.3%)

Despite obtaining advanced training in education, the majority of fellow respondents (71.4%; 5 out of 7) reported that they rarely read MedEd journals independently. Approximately half of the fellows (57.1%; 4 out of 7) indicated that they had published zero MedEd abstracts in the prior year, and only 14.3% had published a MedEd research article over that time period (tertiary objective 2, Table 4).

**Table 4:** MedEd scholarship reported by fellows Each box indicates the number of respondents out of the total number of respondents who selected that Likert scale score. The fraction is also shown as a percentage. MedEd, medical education; N/A, not applicable.

Fellow Survey Question		0	1-3	4-6	7-9	≥10
How many MedEd research abstracts would you estimate you have published in the last year?		4/7 (57.1%)	3/7 (42.9%)	N/A	N/A	N/A
How many MedEd research articles have you published in the past year?		6/7 (85.7%)	1/7 (14.3%)	N/A	N/A	N/A

All (100%; 8/8) faculty respondents indicated that there was no dedicated MedEd journal club run by their hospital, department, or medical school. Only one faculty (12.5%; 1 out of 8) indicated that they reviewed MedEd journal articles with their fellows on a regularly scheduled basis. The majority of faculty respondents (75%; 6 out of 8) indicated that the CORD MEJC was a valuable supplement to the scholarly component of their fellowship program, was a valuable way to build a virtual CoP, and led to networking opportunities otherwise unavailable for their fellows (primary objective, Table 5).

**Table 5:** Faculty perceptions of the CORD MEJC Likert scale (1-5): 1 = strongly disagree, 2= disagree, 3 = neither agree nor disagree, 4 = agree, and 5 = strongly agree. Each box indicates the number of respondents out of the total number of respondents who selected that Likert scale score. The fraction is also shown as a percentage. CoP, community of practice; CORD, Council of Residency Directors; MEJC, Medical Education Journal Club; N/A, not applicable.

Faculty Survey Question	Likert Scale Score				
	1	2	3	4	5
The CORD MEJC content was a valuable supplement to the scholarly component of my fellowship program	N/A	N/A	2/8 (25%)	N/A	6/8 (75%)
The CORD MEJC content was a valuable way to build a virtual CoP to supplement my fellowship program	N/A	N/A	2/8 (25%)	N/A	6/8 (75%)
The CORD MEJC sessions have led to networking opportunities otherwise unavailable for my fellows	N/A	N/A	3/8 (37.5%)	2/8 (25%)	3/8 (37.5%)

**Figure 1:**
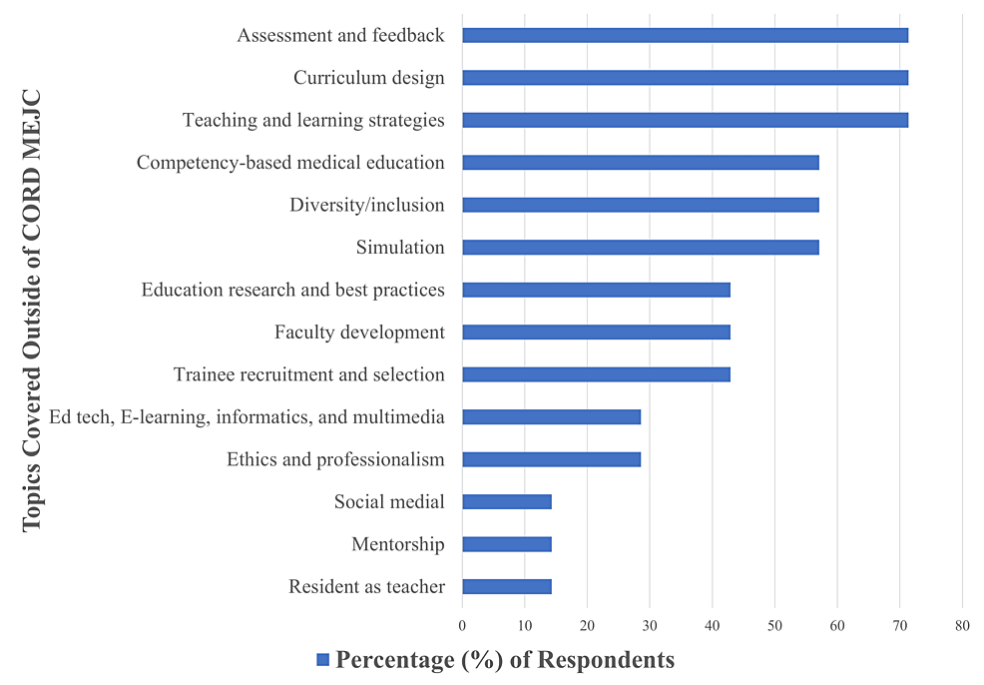
MedEd topics covered by fellowship programs outside of the CORD MEJC CORD, Council of Residency Directors; MedEd, medical education; MEJC, Medical Education Journal Club.

Fellows indicated that they cover a large number of MedEd topics outside of the CORD MEJC in their regularly scheduled fellowship curricula. The most popular topics included assessment and feedback, curriculum design, and teaching and learning strategies (71.4% of respondents), followed by competency-based MedEd, diversity and inclusion, and simulation (57.1% of respondents). Topics with infrequent coverage included residents as teachers, social media, and mentorship (reported by only 14.3% of fellows) (Figure 1). There was a great deal of overlap between topics covered in the MEJC sessions compared with those covered by fellowship programs outside of the MEJC (tertiary objective 1, Figure 1, Table 1). The least frequently covered topics within fellowship programs included education technology, e-learning, informatics, and multimedia; ethics and professionalism; social media; mentorship; and residents as teachers (Figure 1). Two of these topics were covered in MEJC sessions: informatics (during the “Learning Analytics” session) and ethics (during the “Deconstructing Racism” session) (tertiary objective 1, Table 1).

Challenges preventing faculty attendance to CORD MEJC sessions included the timing of sessions, competing priorities, and lack of knowledge of the program. Fellows likewise indicated difficulties coordinating attendance with their shift schedules and lack of knowledge of the program as attendance barriers. Faculty and fellows also noted that poor advertising and technology difficulties (i.e., failure to receive CORD listserv emails) prevented their registration and attendance (Table 6).

**Table 6:** Free response questions *Note that there are no limitations on the number of participants per session. CORD, Council of Residency Directors; MEJC, Medical Education Journal Club.

Survey Question	Respondent Group	Free Response Answers
What challenges served as barriers to you attending CORD MEJC sessions this year?	Faculty	“Everything, including meeting time, need to do other work with fellows, med school responsibilities, fellows post-overnight on journal club day, our own residency conference”
		“Didn't know it existed until attending CORD, and then it appears that there's a limited list for sign-ups”*
		“Thursdays are sometimes difficult to request off, but I'm not suggesting a change - it simply is difficult to coordinate schedules across all our programs”
		“Timing of events”
	Fellows	“Conflicting shifts and travel (flights)”
		“Variable timing; having other fellowship and personal obligations making another shift request difficult”
		“Timing, but that was mainly due to my own error in not requesting those dates off”
		“I could never get added to the email listserv for reminders”
		“I didn't know about them until now!”
		“Work”
Describe ways in which you utilized information from the journal club to improve your scholarly work in medical education research	Fellows	“Used some of the principles to assist in writing an abstract I am working on”
		“I think the remediation session helped me re-think strategies with particular residents. The analytics session made me think about cross-collaboration more”
		“Networking with other medical education fellows and faculty was helpful”
		“I was able to use the information from the journal club this year to write a resolution that passed for EMRA. I have not yet published other work in medical education research”

## Discussion

This PDSA QI survey study found that the CORD MEJC has reached MedEd fellows and faculty at 10 academic institutions across all four regions of the United States. The majority of faculty and fellow respondents found the program to be a helpful supplement to their fellowship curricula, improving access to networking opportunities for fellows (our primary objective). Our results can be extrapolated to indicate that the CORD MEJC is filling a perceived need for building a national MedEd virtual CoP (tertiary objective 3). First, the CORD MEJC created a virtual CoP, which creates a potentially robust network in a landscape that has relatively few programs with small numbers of fellows at each program. Nearly all respondents agreed that this series afforded networking opportunities not previously available at their individual fellowships. Establishing these types of communities of practice has been shown to lead to scholarship, novel educational programming, and leadership positions, all of which are valuable to burgeoning clinician educators [2,6].

There were mixed data regarding whether the CORD MEJC improved fellow knowledge, attitudes, and skills surrounding MedEd research (our secondary objective) and whether the CORD MEJC improved fellows’ ability to describe how to develop scholarly work within MedEd (tertiary objective 2). Subjective data collected from fellow respondents (Table 3) suggested that 57.1% of fellows felt that they had developed the skills they needed to perform MedEd research, and 42.9% of fellows felt that their knowledge about and attitude toward MedEd research had improved. This seemed discrepant with the fact that 57.1% had published zero MedEd abstracts in the prior year, and only 14.3% had published a MedEd research article over that time period. It is unclear why these subjective perceptions and objective results were not in alignment, though it is possible that survey participants were in the process of producing scholarly work that simply had not been published at the time of survey dissemination. The outcome assessment of MedEd fellowship graduates states that graduating fellows have nearly five peer-reviewed MedEd research publications [3]. In the future, we identified the direct generation and dissemination of MedEd scholarship as an area for improvement in the CORD MEJC curriculum. As part of the “Act” portion of the PDSA cycle, our first planned session for the 2023-2024 academic year was a MedEd research panel with experts in the field, during which fellows had the opportunity to learn more about the logistics of publishing scholarship within MedEd. We also plan to conduct a follow-up survey at the end of the 2023-2024 academic year to capture the pending publications to address the discrepancy described above.

Fellow respondents indicated that residents as teachers, social media, and mentorship were minimally covered in their fellowship curricula outside of the CORD MEJC. Though volunteer presenters select their own topic for the MEJC, as coordinators we will request inclusion of these topics (or interweaving within other topics) during upcoming sessions for the 2023-2024 academic year. However, the inclusion of these topics will be balanced by the aforementioned goals of the CORD MEJC curriculum.

Limitations

Our response rate was low, and while we aimed to reduce sampling error by inviting our entire population, this low response rate raises the potential for nonresponse bias, as those who chose not to respond may have different opinions about the MEJC than those who did. The limited number of responses, as well as the self-reported nature of the data, limits our ability to draw definitive conclusions about our study objectives. Another limitation is the fact that the number of participants in attendance at each MEJC session was not recorded. Despite these limitations, this article will serve as a needs assessment for continued curriculum development, as capturing the practices of 22% (10 out of 45) [3,11] of MedEd fellowship programs yields important information about the value and next steps for MEJC. Additionally, because we chose to survey fellowship directors and fellows, we attempted to minimize the possibility of coverage error by querying different stakeholders. Additionally, while Likert scales are inherently limited in their ability to assess program impact, this survey provides valuable information as the MEJC continues to iterate in the PDSA cycle. Future studies could evaluate the scholarly productivity of CORD MEJC participants compared with non-participating MedEd fellows to determine whether our program reaches higher levels of impact on Miller’s pyramid (i.e., “shows how” and “does”) [12].

An objective that was not met by our CORD MEJC during the 2022-2023 academic year was determining whether we broadened fellow exposure to key topics within MedEd (tertiary objective 1). Our survey asked fellows which of our MEJC sessions they attended and which MedEd topics they covered outside of the MEJC as part of their fellowship curriculum. To answer our tertiary research objective more effectively, we should have asked fellows, “Which topics did you learn about this year in CORD MEJC sessions that were not covered in your fellowship curriculum?” We plan on adding this question to the 2023-2024 CORD MEJC QI survey.

One potential limitation of this study is the fact that fellows (who are not yet experts in MedEd) presented the journal club sessions. Best practice recommendations from the CORD for EM residencies suggest that faculty members should provide mentorship for residents when selecting topics and articles for journal clubs [13]. There are no best practice recommendations for MedEd fellowship journal clubs, but guidance would likely be similar. A MedEd expert approved of the topics suggested by fellows, and fellows were encouraged to work with their local fellowship leadership when developing their presentations. During future iterations of the journal club, fellows will be instructed to seek guidance from their fellowship directors when selecting topics for presentation. Structured appraisal tools for evaluating the session articles are also recommended in the CORD best practice guidelines [13]. These tools have been demonstrated to improve journal club educational value without creating extra work for those presenting [14-16]. We plan to implement the use of an extant appraisal tool during the 2023-2024 academic year [17].

As indicated by the free response section of the survey (Table 6), both faculty and fellows indicated a lack of knowledge regarding the existence of the CORD MEJC until the academic year was nearing its close, as well as frustrations with poor amplification and dissemination of journal club articles. The fact that respondents indicated limited knowledge of the program's existence could partially explain our low survey response rates. This feedback from the “Study” portion of our PDSA cycle will be utilized to improve advertising efforts for the 2023-2024 academic year. Specifically, we announced our introductory CORD MEJC session to the entire CORD listserv rather than isolating this announcement to the CORD MedEd Fellowship CoP. We also intend to utilize social media to advertise our program and specific MEJC sessions over the coming year as part of the “Act” portion of the PDSA cycle.

## Conclusions

Here we describe the results of a survey-based QI assessment of the first national MedEd fellowship journal club, the CORD MEJC, over the 2022-2023 academic year. Though the response rate was low, the survey captured the practices of nearly a quarter of existing EM MedEd fellowships in the United States. Survey results indicate that our journal club has helped to foster a virtual CoP for fellows, creating networking opportunities that would be otherwise unavailable to them in their fellowships. We hope to use the data from this study to systematically improve the CORD MEJC, focusing on helping MedEd fellows increase their research output and exposing them to key MedEd topics to which they are not receiving exposure in their fellowships.
